# Decision-Making on Nicotine Replacement Therapy Use and Product Selection: An Explorative Qualitative Study Among Chinese Americans Who Smoke

**DOI:** 10.3390/ijerph23030372

**Published:** 2026-03-16

**Authors:** Nan Jiang, Jennifer Yang, Sue A. Kaplan, Erin S. Rogers, Janice Y. Tsoh, Joanne Chen Lyu, Scott E. Sherman

**Affiliations:** 1 Department of Population Health, NYU Grossman School of Medicine, New York, NY 10016, USA; jy3286@nyu.edu (J.Y.); sue.kaplan@nyulangone.org (S.A.K.); erin.rogers@nyulangone.org (E.S.R.); scott.sherman@nyulangone.org (S.E.S.); 2 Department of Psychiatry and Behavioral Sciences, University of California, San Francisco, CA 94143, USA; janice.tsoh@ucsf.edu; 3 TSET Health Promotion Research Center, Stephenson Cancer Center, University of Oklahoma Health Campus, Oklahoma City, OK 73104, USA; joanne-lyu@ou.edu; 4 Department of Family and Preventive Medicine, College of Medicine, University of Oklahoma Health Campus, Oklahoma City, OK 73104, USA; 5 Department of Medicine, VA New York Harbor Healthcare System, New York, NY 10010, USA

**Keywords:** cigarette, smoking, cessation, nicotine replacement therapy

## Abstract

**Highlights:**

**Public health relevance—How does this work relate to a public health issue?**

- Chinese Americans who smoke have low use of nicotine replacement therapy (NRT).
- This study identified barriers to NRT use and decision-making around NRT product choice among Chinese Americans who smoke and received NRT.

**Public health significance—Why is this work of significance to public health?**

- The study focuses on an understudied population with low uptake of NRT.
- Findings highlight both culturally specific barriers (e.g., skepticism toward pharmacotherapy) and general barriers (e.g., lack of readiness to quit) to NRT use.

**Public health implications—What are the key implications or messages for practitioners, policy makers and/or researchers in public health?**

- Targeted efforts are needed to strengthen NRT education among Chinese Americans who smoke.
- Cessation programs targeting this population should address cultural beliefs about medication, reframe perceptions about willpower, engage those not ready to quit, and provide diverse NRT options with guidance on side effect management and integration into daily routines.

**Abstract:**

Chinese Americans who smoke have low use of nicotine replacement therapy (NRT). This study explored perceptions of NRT and decision-making around product choice among Chinese American smokers who received NRT. From September 2023 to January 2024, we conducted in-depth phone interviews with 20 participants recruited in New York City from a WeChat-based cessation pilot trial and a community-based cessation program, both providing free nicotine patches, gum, or lozenges. Participants (aged 26–72; 85% male; 60% current smoking) included 12 consistent NRT users (≥2 weeks), four trial users (<2 weeks), and four non-users. Five participants (25%) had never heard of NRT before program enrollment, and 14 (70%) had never used it previously. Consistent users generally viewed NRT as helpful in reducing cravings. Others reported barriers, including culturally rooted skepticism toward pharmacotherapy, preference for unassisted quitting, lack of readiness to quit, prior negative experiences, and unpleasant taste or side effects. Product choice was influenced by lay knowledge, ease of integrating NRT into daily routines, perceived effectiveness, and taste and side effects. Cessation programs addressing cultural beliefs, reframing willpower, engaging individuals not ready, and providing diverse NRT options with guidance on side effect management and routine integration may increase NRT use among this population.

## 1. Introduction

Nicotine replacement therapy (NRT) is the most commonly used pharmacotherapy for smoking cessation approved by the U.S. Food and Drug Administration (FDA) [1]. Despite its wide availability [2,3], studies among English-speaking individuals who smoke have identified numerous barriers to uptake and consistent use. These include preference for unassisted quitting; concerns about safety and addictiveness; skepticism regarding effectiveness; prior negative experiences; discouraging peer narratives; inconvenience; and product-related factors such as cost, side effects, and unpleasant taste [4,5,6,7,8,9,10,11].

Little is known about barriers to NRT use among Chinese Americans who smoke, a population with low use of cessation pharmacotherapy [12,13,14,15,16]. Chinese Americans, the largest Asian American ethnic group in the U.S., have an estimated smoking prevalence of 24–34% among men and 2–4% among women [13,14,15,17,18]. Although 47–71% of Chinese Americans who smoke report making at least one quit attempt in the past year, only 12–14% of those attempting to quit use NRT or other cessation pharmacotherapy [13,14,15]. Notably, these estimates are from studies published in the early 2000s, highlighting limited recent data for Chinese Americans. The underuse of NRT is attributed to several barriers, including limited awareness and concerns about safety and effectiveness [16,19,20]. In our recent study in New York City (NYC), one-third of licensed retail pharmacies in neighborhoods with large Chinese American populations did not stock over-the-counter NRT (patch, gum, lozenge), suggesting a potential access barrier [21].

However, when access barriers are reduced through providing free NRT along with information addressing common concerns, it remains unclear how Chinese Americans who smoke decide whether to use NRT and which form to select. Evidence indicates that combining long-acting NRT (patch) with short-acting forms (e.g., gum, lozenge) is more effective than using a single product [22], yet little is known about how individuals evaluate and choose among available NRT products.

To address these gaps, we conducted in-depth interviews with Chinese Americans who currently smoke or recently quit, all of whom had received free nicotine patches, gum, or lozenges through smoking cessation programs. By focusing on individuals who had already obtained NRT, this study sought to examine factors that persist to impact NRT use beyond access barriers that have been well documented in prior research. We also explored decision-making around NRT form selection. Findings may inform culturally tailored strategies to increase cessation pharmacotherapy use in this population, thereby helping reduce tobacco-related health disparities.

## 2. Materials and Methods

### 2.1. Participants and Recruitment

Between September 2023 and January 2024, we recruited 20 participants from two sources: individuals who completed the WeChat Quit Coach pilot trial [23] and individuals who completed a cessation program at Asian Americans for Equality (AAFE), a community-based organization in NYC. Inclusion criteria included: self-identified Chinese American; aged ≥18 years; had smoked at least 100 cigarettes in their lifetime; current (past 30-day) smoking or quit within the past 24 months; receipt of NRT through one of the programs; and NYC residence.

The WeChat Quit Coach is a 6-week, WeChat-based Chinese-language mobile messaging cessation program for Chinese immigrants who smoke, regardless of the level of readiness to quit [23]. The intervention was designed to increase knowledge of smoking harms, motivation, self-efficacy, and NRT use. Of the 42 intervention messages, two specifically addressed NRT safety, effectiveness, and addictiveness. Participants in both intervention and control groups could request a free 4-week supply of nicotine patches and lozenges by calling or texting research assistants (RAs), who recommended a combination of patch and lozenge based on cigarettes per day and time to first cigarette after waking, and mailed the medications with printed instructions and side effect information.

AAFE’s cessation program, part of NYU Langone Hospitals’ Community Service Plan [24], provides brief in-person counseling to Chinese Americans who smoke with referrals to the Asian Smokers’ Quitline. The program offers up to a 4-week supply of free nicotine patches and gum, along with instructions for use and information on potential side effects.

For recruitment from the WeChat Quit Coach trial, RAs reviewed NRT use data from the 6-month follow-up survey (administered August 2022–May 2023), which included two questions on NRT use: “In the past 6 months, have you used any NRT products?” and “On how many days did you use each NRT product?” For recruitment from AAFE’s program, AAFE staff contacted individuals enrolled between September 2021 and June 2023, asked two questions regarding NRT use (“Since you completed AAFE’s brief cessation counseling, have you used any NRT products?” and “On how many days did you use each NRT product?”), and referred potentially eligible individuals to the study team.

Among 54 eligible individuals from the WeChat Quit Coach trial and 12 from AAFE’s program, we used purposive sampling to recruit 20 participants (14 from WeChat Quit Coach; 6 from AAFE) to ensure a diverse sample by sex and NRT use duration, including consistent use (≥2 weeks of any NRT), trial use (<2 weeks), and non-use (received but did not use NRT). Selected individuals were contacted by phone by a bilingual RA, informed of the study purpose, screened for eligibility, and scheduled for interviews. All contacted individuals agreed to participate. The study was approved by the Institutional Review Board of NYU Grossman School of Medicine (i23-00949).

### 2.2. Interview Procedures

Two bilingual team members (NJ and JY) conducted the interviews by phone. Verbal informed consent was obtained prior to the interview. A semi-structured interview guide was used to explored three primary domains: (1) reasons for NRT use or non-use (e.g., “What led you to request NRT?” “Why did you decide to use (or not use) NRT?”); (2) perceptions of NRT, including barriers and facilitators to uptake and consistent use (e.g., “What did you like or dislike about NRT?” “Did you find NRT helpful? Why or why not?” “Would you use NRT again in your future quit attempt? Why or why not?”); and (3) decision-making regarding product selection (e.g., “When offered both patches and lozenges (or gum), how did you decide which product to use?”). Probes were used throughout the interviews to clarify responses and/or elicit more depth, nuanced insights. Interviews were conducted in Mandarin or Cantonese, lasted 12–30 min, and were audio-recorded. Each participant received a $20 gift card by mail.

### 2.3. Data Analysis

Audio recordings were transcribed verbatim in Chinese and verified for accuracy. Two bilingual team members (NJ and JY) used both deductive (based on interview guide domains) and inductive (open coding) approaches [25,26] to code the transcripts using NVivo 12 [27]. An initial codebook was developed from the interview guide and included code definitions. The two coders independently coded an initial subset of transcripts and then met to compare coding, resolve discrepancies, and refine the codebook by incorporating emergent codes reflecting salient concepts, collapsing overlapping codes to reduce redundancy, and clarifying code definitions. This iterative process continued until consensus was achieved and the codebook was finalized. The coders then independently coded all transcripts using the finalized codebook and collaboratively identified themes and subthemes through discussion.

The coders monitored data saturation throughout the analytic process. No new codes emerged after approximately half of the interviews had been coded, indicating that saturation had been achieved. This is consistent with empirical evidence showing that data saturation in qualitative research is often reached within 17 or fewer interviews, particularly in relatively homogeneous samples with focused study objectives [28,29,30,31].

## 3. Results

Participants (26–72 years) were mostly male (85%; Table 1). Twelve (60%) reported current smoking and eight (40%) had recently quit. For NRT use, 12 participants (60%) reported consistent use (six currently smoking, six had quit), four (20%) reported trial use (1–2 days) (two currently smoking, two had quit), and four (20%) reported no use (all currently smoking).

### 3.1. Limited Awareness and Prior Use of NRT

Five participants (25%) reported they had never heard of NRT before enrolling in the cessation programs. Fourteen (70%), including those five, had never used NRT prior to program enrollment.

*“I’d heard of nicotine gum before, and I knew it was for quitting smoking. But I didn’t really know what it was. Since AAFE offered it for free, I decided to give it a try.”*

(P1, female, consistent user)

### 3.2. Motivation for NRT Use

Consistent users often reported using NRT to support quitting: *“I’d been trying to quit for a while. AAFE gives out NRT for free, so I got it and gave it a try”* (P4, male, consistent user). One consistent user described smoking reduction as the primary goal: *“I kinda wanted to quit, but mainly I just wanted to cut down”* (P8, male, consistent user). By contrast, trial users typically cited curiosity as their motivation, with no intention to quit or reduce smoking: *“I was just curious about it [lozenge], as I’d never used it before”* (P11, female, trial user).

### 3.3. Barriers to NRT Use

Five themes emerged as barriers to uptake and consistent use: (1) culturally rooted skepticism toward pharmacotherapy; (2) preference for unassisted quitting; (3) lack of readiness to quit; (4) prior negative experiences; and (5) unpleasant taste or side effects.

#### 3.3.1. Culturally Rooted Skepticism Toward Pharmacotherapy

Several participants across sex and age groups referenced the common Chinese saying, *“All medicine carries some degree of poison.”* This deeply ingrained cultural belief shaped the perception that medications should be avoided unless necessary, leading participants to believe that quitting without pharmacological support was preferable.

*“My family and I try to avoid taking medicine whenever we can. Last winter, my daughter caught a cold and her pediatrician prescribed medicine. I gave her half the recommended dose.”*

(P11, female, trial user)

*“I avoid taking medicine when possible. Even if I get a fever, I just rest and rely on my own immune system to recover.”*

(P20, male, non-user)

#### 3.3.2. Preference for Unassisted Quitting

Many participants regarded unassisted quitting as their first choice, viewing NRT as unnecessary or a backup option. This preference was often rooted in the belief that willpower is the key and sufficient to achieve abstinence. For these participants, quitting unaided was viewed as a demonstration of self-discipline, whereas using cessation aids as a sign of weak determination.

*“I want to try quitting on my own. It’s not that I’m against NRT, but if I can’t quit on my own, if my willpower doesn’t work, I’d have no choice but to use NRT.”*

(P15, male, non-user)

*“It [quitting] depends on how determined you are. Medicine only helps a little. Like my friend, once he made up his mind, he just quit without using anything.”*

(P20, male, non-user)

For some, especially those who smoked nondaily, this preference was often related to denial of nicotine dependence, leading them to dismiss the need for cessation aids.

*“I don’t smoke much, just one or two cigarettes a day, and not even every day. I’m not addicted, so I don’t think I need NRT.”*

(P11, female, trial user)

#### 3.3.3. Lack of Readiness to Quit

Eleven participants reported not yet being ready to quit. Among them, two requested NRT for future use once they feel ready.

*“I still have a few cartons [cigarettes] left in my drawer. I plan to quit after I finish those. Having NRT on hand makes me feel more confident about quitting.”*

(P14, male, non-user)

#### 3.3.4. Prior Negative Experiences

Six participants had previously used NRT. Among them, two reported experiencing unpleasant side effects or perceived ineffectiveness, which made them reluctant to use it again.

*“About eight years ago, I tried the patch. It made me dizzy and groggy… I thought it would help control cravings, but it didn’t. So I never used it again”*

(P12, male, non-user)

#### 3.3.5. Unpleasant Taste or Side Effects

One participant discontinued NRT after experiencing adverse effects during the trial. Unpleasant taste and side effects hindered continued use.

*“The patch made my skin itchy. I ripped it off within 30 minutes of putting it on. I couldn’t stand it… The lozenge made me gag and feel nauseous. I couldn’t take it.”*

(P17, male, trial user)

### 3.4. NRT Form Selection

While participants were offered both nicotine patches and lozenges (or gum), only three (15%) reported consistent use of both products. Many initially tried both forms, but ultimately chose one. Product selection was influenced by four key factors: (1) lay knowledge, (2) ease of integration into daily routines, (3) taste and side effects, and (4) perceived effectiveness.

#### 3.4.1. Lay Knowledge

With limited awareness and prior use, participants generally had an incomplete understanding of how NRT works. They chose products relied on lay knowledge and personal assumptions about safety.

*“I used the patch because it’s applied outside the body, just sticks on the skin. The lozenge, you got to swallow it. That may hurt kidneys and other organs. Between the two, the patch is safer.”*

(P16, male, consistent user)

*“I’m scared of the lozenge because I’ve never heard of it.”*

(P18, female, trial user)

#### 3.4.2. Ease of Integration into Daily Routines

Participants discussed how convenience and compatibility with daily life influenced product choices. Some favored the patch for its simplicity, while others preferred lozenges or gum for their flexibility and on-demand use.

*“I work on a food cart. It’s hot and I sweat a lot. The patch just doesn’t work. It makes me feel itchy.”*

(P9, male, consistent user)

*“I like the patch. It’s easy, just put it on before going to bed… With lozenges, I have to remember to carry them around.”*

(P10, male, consistent user)

*“The gum is so convenient. Whenever I need it, I just pop a piece.”*

(P1, female, consistent user)

#### 3.4.3. Taste and Side Effects

Participants reported side effects, such as skin irritation, nausea, dizziness, mouth or throat soreness, heart palpitations, and anxiety. Several also noted unpleasant taste or discomfort with product size. These experiences influenced product selection and sometimes led to switching or early discontinuation.

*“The gum tastes like chewing wax, like gnawing on tree bark… It’s bitter.”*

(P4, male, consistent user)

*“The regular-size lozenge is too big. It’s uncomfortable to have such a big pill in mouth…”*

(P6, male, consistent user)

#### 3.4.4. Perceived Effectiveness

Participants’ perceptions of how well a product reduced cravings and relieved withdrawal symptoms played a critical role in product selection. For some, perceived effectiveness outweighed unpleasant taste and side effects.

*“Even though the gum is like chewing wax, it really works. I cut down by half in less than 10 days.”*

(P4, male, consistent user)

*“The patch didn’t do anything, so I stopped after two tries. The lozenge eased my cravings… It gave me heart palpitations, like after coffee, but manageable.”*

(P7, male, consistent user)

### 3.5. Perceptions of NRT

Consistent users generally viewed NRT as helpful, emphasizing its role in reducing cravings, easing withdrawal symptoms, and increasing confidence to quit. Several indicated they would use it again in future quit attempts. In contrast, trial users provided limited feedback due to minimal exposure.

*“They [patches and gum] did help reduce cravings to smoke.”*

(P3, male, consistent user)

*“The gum mostly helped mentally, giving me confidence to quit.”*

(P2, male, consistent user)

*“I can’t tell if it works. I only tried the lozenge once.”*

(P11, female, trial user)

## 4. Discussion

This study examined factors influencing NRT use and product selection among Chinese Americans who smoked and had already received NRT. A notable finding was the limited awareness and prior experience with NRT: 25% of participants had never heard of NRT and 70% had never used it before enrolling in cessation programs. These results align with prior research reporting low awareness of NRT in this population [16,19,20]. Even after receiving brief information on NRT safety and effectiveness with printed instructions on proper use, it appeared that knowledge gaps persisted. Some participants continued to rely on lay knowledge to guide product selection. These findings highlight the need for targeted interventions to strengthen NRT education among Chinese Americans. Tobacco treatment programs and healthcare providers cannot assume familiarity with NRT, but instead provide education on product types, mechanisms of action, and evidence of safety and effectiveness. Public health campaigns targeting this population should promote NRT as an evidence-based cessation aid to increase awareness.

Skepticism toward pharmacotherapy, deeply rooted in Chinese cultural beliefs, emerged as a major barrier to NRT use. Consistent with prior research [19], participants often expressed that medications should be avoided unless absolutely necessary, reflecting entrenched safety concerns. This belief shaped the perception that quitting without pharmacological aid was preferable, leading many to dismiss NRT as a viable cessation aid. Addressing these cultural beliefs is essential to promoting NRT use. One potential strategy is to emphasize the relative safety of NRT compared with cigarettes, underscoring that NRT delivers nicotine without the harmful toxicants produced by cigarettes and that using NRT to support abstinence is substantially safer than continued smoking.

The preference for unassisted quitting also emerged as a key barrier. This preference was often grounded in the belief that willpower alone is sufficient to achieve abstinence. For these individuals, unassisted quitting was perceived as reflective of self-reliance and discipline, values that are central in Chinese cultural contexts. In contrast, using cessation aids was sometimes viewed as a sign of weak determination. Prior studies have documented willpower beliefs among both Chinese-speaking [16,19,20,32,33,34] and English-speaking populations [7,9,10,11,35,36]. Individuals endorsing these beliefs often regard quitting as a personal responsibility rather than an endeavor that can be supported by external aids [11,20]. To address this barrier, public health campaigns and cessation programs need to emphasize that, although willpower is important, it is often unsustainable, which makes it difficult to quit by relying on willpower alone. Messaging must clarify that medications do not substitute for willpower but instead complement it by alleviating withdrawal symptoms, and that personal efforts including self-discipline remain essential for behavioral changes in quitting.

Consistent with prior research [8,10,37], lack of readiness to quit also impeded NRT uptake. The commonly held belief that one must be fully committed before it is worthwhile trying to quit may discourage people from taking action. Several participants requested NRT to keep on hand for future quit attempts, reflecting ambivalence rather than rejection and indicating an interest in quitting despite not yet feeling ready. Future interventions should clarify that readiness is not a prerequisite and encourage these individuals to engage in “practice quit attempts” without the pressure of permanent quitting [38], motivating them to initiate the quitting process. Evidence suggests that providing NRT to individuals not yet ready may catalyze experimentation with cessation aids and strengthen motivation. Prior studies showed that NRT sampling among individuals unready to quit increased quit attempts and NRT use [38], and offering free NRT to primary care patients who smoke (regardless of readiness) increased quit attempts, NRT use, and abstinence [39]. Future research is warranted to examine the effects of NRT provision on cessation outcomes among Chinese Americans who smoke and are not ready to quit.

Participants, particularly those who smoked nondaily, often did not view themselves as addicted and therefore perceived no need for pharmacotherapy. This finding aligns with prior qualitative research among Chinese-speaking individuals who smoke [33] and quantitative evidence showing light smoking is associated with unassisted quitting compared to heavier smoking [40]. Cessation programs may help individuals understand that nicotine dependence can be underestimated, which undermines their chance of benefiting from evidence-based aids.

Consistent with prior research [4,6,7,10,37,41], past negative experiences and unpleasant side effects were barriers to NRT use. Product taste, side effects, ease of integration into daily routines, and perceived effectiveness also influenced product choice. Cessation programs may consider offering multiple product options, providing guidance on side effect management, and supporting product selection that fits individuals’ daily routines. Notably, many consistent users in this study reported experiencing side effects and mixed effectiveness, yet still acknowledged NRT’s role in reducing cravings and withdrawal symptoms and expressed willingness to use it again. Given the influence of peer narratives on NRT perceptions and use [4,9,11,20,33,41], incorporating consistent users’ positive testimonials into cessation programs may help increase acceptability, uptake, and consistent use.

This study has several limitations. Participants were recruited from two NYC cessation programs and limited to individuals who had obtained NRT, which may limit generalizability, particularly to individuals who would decline pharmacotherapy or reside in other regions. Nonetheless, prior qualitative research among Chinese Americans who smoke in California has documented similar barriers, suggesting that some findings may extend across U.S. regions [19]. In addition, we did not assess e-cigarette use or perceptions of NRT dosage/strength, both of which may influence NRT uptake and use patterns and warrant further investigation.

Nonetheless, this study has several strengths. First, we focused on an understudied population with low NRT uptake and included participants with varied NRT use patterns (non-use, trial use, and consistent use), providing a comprehensive, nuanced view of factors influencing NRT use and product choice. Second, by recruiting individuals who had already obtained NRT, the study provided insights into product perceptions in a context where key access barriers (e.g., limited awareness and cost) were addressed, thereby highlighting cognitive, cultural, and product-specific factors that persist to impede NRT use even when access is facilitated. Third, the study offered insight into decision-making around NRT form selection and how product-specific factors influence uptake and consistent use.

## 5. Conclusions

This study reveals limited awareness and prior experience with NRT among Chinese Americans who smoke, highlighting the need for targeted education. While consistent users generally perceived NRT as helpful, others reported barriers including culturally rooted skepticism toward pharmacotherapy, preference for unassisted quitting, lack of readiness, prior negative experiences, and unpleasant taste and side effects. Product choice was influenced by lay knowledge, compatibility with daily routines, perceived effectiveness, and product taste and side effects. Awareness campaigns and treatment programs targeting this population should address cultural beliefs, reframe willpower perceptions, and develop strategies to engage those not yet ready to quit. Interventions providing diverse product options with guidance on side effect management and routine integration, and incorporating positive peer testimonials may help enhance NRT use within this population.

## Figures and Tables

**Table 1:** Participant characteristics and use of nicotine replacement therapy.

Participant ID	Sex	Age	Smoking Status at Interview	NRT Use			Prior NRT Use Before Enrolling in Cessation Programs
				Use Duration ^a^	Product Used	Primary Reason for NRT Use	
Participants recruited from the community-based smoking cessation program at Asian Americans for Equality							
P1	Female	55	Former	Consistent use	Gum: 12 weeks	Quitting	No
P2	Male	60	Former	Consistent use	Gum: 2–3 weeks	Quitting	Yes
P3	Male	67	Former	Consistent use	Patch: 2 weeks Gum: 2 weeks	Quitting	No
P4	Male	38	Current	Consistent use	Patch: 4 weeks Gum: 4 weeks	Quitting	No
P5	Male	63	Current	Consistent use	Gum: 8–12 weeks	Quitting	No
P6	Male	72	Former	Consistent use	Patch: 4 weeks Lozenge: 1 week Gum: 1 day	Quitting	Yes
Participants recruited from the pilot trial of the WeChat Quit Coach intervention							
P7	Male	30	Former	Consistent use	Patch: 2 days Lozenge: 8 weeks	Quitting	No
P8	Male	39	Current	Consistent use	Patch: 2 days Lozenge: 4 weeks	Smoking reduction	No
P9	Male	30	Current	Consistent use	Patch: 1 day Lozenge: 12 weeks Gum: 1 day	Quitting	Yes
P10	Male	60	Former	Consistent use	Patch: 3–4 weeks Gum: 2 days	Quitting	Yes
P11	Female	32	Former	Trial use	Lozenge: 1 day	Curiosity to try	No
P12	Male	48	Current	No use	--	--	Yes
P13	Male	40	Current	Consistent use	Patch: 2 weeks	Quitting	No
P14	Male	64	Current	No use	--	--	No
P15	Male	57	Current	No use	--	--	No
P16	Male	42	Current	Consistent use	Patch: 3–4 weeks	Quitting	No
P17	Male	37	Former	Trial use	Patch: 1 day Lozenge: 1 day	Quitting	No
P18	Female	26	Current	Trial use	Patch: 1 day	Curiosity to try	Yes
P19	Male	33	Current	Trial use	Patch: 2 days	Curiosity to try	No
P20	Male	40	Current	No use	--	--	No

NRT: nicotine replacement therapy. ^a^ NRT use duration: Use of any NRT product for ≥2 weeks were regarded as consistent use; <2 weeks as trial use; and 0 days as no use.

## Data Availability

The raw data supporting the conclusions of this article (i.e., qualitative interview transcripts in Chinese) will be made available by the authors upon request.

## Abbreviations

The following abbreviations are used in this manuscript:

NRT	Nicotine replacement therapy
FDA	Food and Drug Administration
NYC	New York City
AAFE	Asian Americans for Equality
RA	Research assistant
